# Evaluation of pectin extractions and their application in the alkaline Maillard reaction

**DOI:** 10.1038/s41598-022-22002-9

**Published:** 2022-11-18

**Authors:** María-Guadalupe Guízar-Amezcua, Alvaro Pineda-Santana, Martha-Isabel González-Domínguez, Leonardo-Ramses Cajero-Zul, Luis-Guillermo Guerrero-Ramírez, Armando López-Miranda, Apolo Nambo, Janneth López-Mercado

**Affiliations:** 1 Departamento de Ingeniería de Industrias Alimentarias, Instituto Tecnológico de Estudios Superiores de Zamora, Km 7 carretera Zamora-La Piedad S/N, Col, El Sauz de Abajo, C.P. 59720 Zamora, Michoacán México; 2 Ingeniería en Nanotecnología, Universidad de la Ciénega del Estado de Michoacán, Lomas de la Universidad, Avenida Universidad No. 3000, C.P. 59103 Sahuayo, Michoacán México; 3 Centro Universitario de Ciencias Exactas e Ingenierías, Universidad de Guadalajara, Blvd. Marcelino García Barragán No 1421, C.P. 44430 Guadalajara, Jalisco México; 4 Dirección de Materiales de Referencia, Centro Nacional de Metrología, Carretera a los Cues Km 4.5, El Marques, 76246 Queretaro, Mexico; 5 Conn Center for Renewable Energy Research, University of Louisville, Louisville, KY 40292 USA

**Keywords:** Biotechnology, Structural biology, Environmental sciences, Chemistry, Energy science and technology, Engineering

## Abstract

A 2^3^ factorial design was used to evaluate the influence of temperature, catalyst and time and esterification degree (DE) of pectin obtained from mango, orange and tangerine peels as well as tamarind seeds by using the acid hydrolysis method. The study showed that a high temperature positively influenced the percentage of pectin yield for the four second generation biomasses. Nevertheless, the temperature showed a greater influence in the solubility and diffusion of the acid solvent in the tamarind seed matrix, resulting a pectin recovery 32.9%. Concerning the %DE, the most statistically significant value observed was dependent on the type of biomass studied. The %DE and the nature of the pectin are determining factors in the pectin’s final use, in the present work the pectin extracted was used to produce furfural, a precursor of high value chemicals. The furfural production was achieved through alkaline hydrolysis and enhanced using the Maillard reaction, reaching a maximum concentration of 71.8 g/L which represents a 42.1% increase from the alkaline hydrolysis.

## Introduction

The food industry has an enormous market with a great variety of processes involved, generating vast amounts of waste^1^ rich in bioactive macromolecules such as pectin, which are high-added value compounds^2^. Within these large waste volumes, the highest quantities of ready to use biomass. Particularly the seeds and peels are of major interest since they contain lower moisture content than the original fruit^3^. Citrus fruits such as orange and tangerine have great importance due to their high production in the world^4^ and, specially, in Mexico^5^. Cypriano et al.^6^ (2018) reports an orange production worldwide of 50.2 million tons in 2017. America is responsible for more than 50% of the world´s production of oranges, with Mexico as the leading country^7^. The majority of citrus fruits are processed to obtain their juice for beverages or direct consumption^8,9^, generating considerable waste referred to as second generation biomass, for being feedstocks to non-edible lignocellulosic biomass. The main advantage of reprocessing second-generation biomasses is that they don’t generate a cost when used as raw materials. It has been reported that citrus peels are great source of pectin^4,10–12^, including tamarind seeds^13^ and mango peels^3,14–17^. Many studies have reported different variables in the extraction process of pectin. Particularly, Banerjee et al.^18^ (2016) compared the type of acid and process for pectin extraction from the mango peel. They reported that the HCl showed greater yield with the conventional method (25.2%) when compared to sonification method (14.9%), and a higher yield was obtained when lemon juice was used (27.3%). On the other hand, Tovar et al.^19^ (2019) reported the used of phosphoric acid in the pectin extraction, using orange peel, which generated a pectin yield of 29.37% at 95 °C for 2 h.

Pectin is a structural complex polysaccharide that has a high content of galacturonic acid (AGal)^20–24^ with varying degree of esterification (DE)^3,25^. The pectin market was valued in $964.1 million USD in 2015 and projected to grow at a rate of 7.1% for the next 9 years, and Mexico has a big participation in such market. Pectin can be used in food industry^26^ as a gelling agent, thickener, texture enhancer and stabilizing agent^27–31^. Other applications include biofilm production^32–34^, pharmaceutical and cosmetic industry applications^25,35^ or water desalination applications^36^. Pectin has been reported as precursor for chemicals like bioethanol or furanic compounds, such as furfural^12^, which is considered a platform chemical for high value compounds^37^. Furfural can be obtained through pectin hydrolysis using a catalyst such as sulfuric acid, other homogeneous acid catalysts, heterogeneous acid catalysts or even complex reactions such as the Maillard reaction as described in our previous work^3^.

In order to determine if certain pectin is suitable for a specific application, DE needs to be evaluated. DE determines the functional properties of the polysaccharide^20,38^. Pectin is classify as high DE pectin when its DE was above 50%, and bellow this value it is considered a low DE pectin^21,22,29,39^.

DE depends highly on the extraction method, conditions used and raw material. The three most reported methodologies for pectin extraction are microwave^4,21^, ultra-high pressure^40^ or traditional heating^16,40^. In the presence of organic or mineral acids^33,41,42^, in this case, when organic acids are used it is required higher concentrations, higher temperatures and/or longer extractions times. Although mineral acids like HCl or H_2_SO_4_ are cheaper that organic acids^43^ their use requires additional steps to remove toxic compounds when pectin is intended for food industry applications^41^.

In the present work, a detailed experimental design on the traditional heating methodology using mineral acids was developed. Focusing on time, mineral acid type and temperatures during extraction and their impact on the DE and pectin yield among 4 raw materials. This study implements the use of non-conventional raw materials as mango peels (*Manguifera Indica L*.), mandarin peels (*Citrus reticulada L.*) and tamarind seeds (*Tamarindus indica L.*) and compares them with orange peels (C*itrus sinensis*) in the generation of furfural through alkaline hydrolysis and alkaline Maillard reaction.

## Material and methods

Mango kent peels (*Manguifera Indica L*.) were supplied by FREXPORT, S.A. de C.V., orange peels (Ci*trus sinensis*) by AGRANA FRUIT MÉXICO, S.A. de C.V. company. Tamarind seeds (*Tamarindus indica L.*), and mandarin peels (*Citrus reticulada L.*) were obtained from local consumption. Second-generation biomasses were dried out to avoid microorganism growth during storage. Drying conditions were 60 °C for 24 h. Once dried, samples were ground and sieved through a no. 18 mesh to obtain homogeneous powders with particle sizes of about 1 mm. The moisture content of agroindustrial residues were determined using a Denver Instrument thermobalance model IR 120 at 105 °C until constant weight^39^.

### Biomass characterization

The second-generation biomasses were characterized through scanning electron microscopy (SEM) and Energy-dispersive X-ray Spectroscopy (EDS). The morphology and diameter were determined by micrograph analysis, while the elemental analysis was determined using Energy Dispersive X-Ray Spectroscopy (EDS). SEM analysis was performed by using JEOL JSM-6610-LV Electronic Microscope (JEOL, Tokyo, Japan) operated at 15 kV and working distance of 10 mm. The magnifications of SEM images were 100×, 500× and 1500×. For the EDS analysis, two zones (area) on different surface of the biomasses were taken.

### Pectin extraction

The acidic hydrolysis methodology employed for the pectin extraction is reported elsewhere^11^, and involves the use of a 0.03 mass to volume ratio of biomass to aqueous solution, adjusting the acidic solution pH = 2 under continuous stirring. In this work a 3-factor 2-level (2^3^) experimental design was employed in each biomass (Table 1) in order to understand and determine the role of the variables involved in the pectin extraction through the acidic hydrolysis.

**Table 1:** Operating variables values used in the 2^3^-factorial design studies for each biomass in the pectin extraction process.

Coded variable	Operating variable	Low level (−)	High level ( +)
A	Catalyst type (qualitative)	HC1	H_2_S0_4_
B	Extraction time (min)	30	60
C	Extraction temperature (°C)	60	80

The pectin extraction methodology used in this work consist of the following steps: (1) biomass drying at 60ºC until constant weight was obtained, (2) biomass hydrolyzation followed the experimental design described in Table 1, (3) hydrolyzed pectin separation, during this step the hydrolyzed pectin was separated from the rest of the biomass through centrifugation (500 rpm for 20 min) and precipitation (12 h) by adding an equal volume of ethanol (98%), (4) pectin filtration and (5) pectin drying at 50ºC until constant weight was obtained. Finally the dried pectin obtained was analyzed to determine the pectin yield, degree of esterification (DE), morphology and elemental content. These results were correlated to the variables involved in the experimental design applied in this study.

### Pectin characterization

ATR-FTIR analyses were carried out using a Nicolet iS50 infrared spectrometer (4000–400 cm^−1^) with 32 scans and 4 cm^-1^ resolution. Spectra were obtained by duplicate and the bands corresponding to the esterified carboxylic groups, COO-R, and to free carboxyl groups, COO- were used to determine the DE, according to Eq. (1)^3,29,42,44^. Pectin DE was calculated using Origin 8.0 software^45,46^.

1 $$ \% DE = \frac{Area\; of\; esterified\; carboxyl \;groups}{{area \;of \;esterified \;carboxyl \;groups\; + \;area \;of \;non \;esterified}} \times 100 $$

Samples were analyzed by scanning electron microscopy (SEM) and Energy-dispersive X-ray Spectroscopy (EDS), were performed using 20 kV in a JEOL JSM6300 LV, to analyze the presence of salts.

Thermal analysis (Thermogravimetry TG) was carried out using a TRIOS V3, under the following conditions: linear heating rate 20 °C/min from room temperature to 600 °C and dynamic inert nitrogen atmosphere (100 mL/min). The samples studied were the pectin from four biomasses at the conditions of Experiment 8 (E8) (Table S1).

### Experimental Statistical Design

The statistical optimization technique using the full experimental factorial design was applied to determine the highest pectin yield Eq. (2) in correlation with the extraction conditions^47^ and to determine their effect in the % DE and salt content.

2 $$ \% Pectin \;yield = \frac{Weight\left( g \right)of \;dry \;pectin}{{Weight\left( g \right)of\; dry\; biomass}}x100 $$

For this study 8 experimental runs were generated for each biomass (Table S1). Each factor was studied at both low and high levels. The higher level was designated as (+) and the lower value was designated as (−) (Table S2). All experiments were performed in duplicate (to approximate the experimental error). The extraction parameters were optimized using analysis of variance (ANOVA). The experiments were carried out and data was statistically analyzed by Pareto diagrams and normal probability of standardized effects to determine the magnitude, direction and importance of the effects. The confidence level for statistical significance was set at a probability value of 0.05.

### Evaluation of pectin in furfural production

Furfural production from mango, orange, tangerine and tamarind pectins was performed through the alkaline hydrolysis using a batch- type reactors containing 2% by weight of pectin in a Ca(OH)_2_ (pH = 10) or CaCl_2_ (pH = 10) solution. The reactors were heated to a constant temperature of 160 °C for 15 min, 30 min, 45 min, 60 min, 90 min and 120 min, with a stirring of 1500 rpm. The Maillard reaction was performed using 1% by weight of pectin and 1% by weight of lysine in the batch reactors using the solution of Ca (OH)_2_ and CaCl_2_, in the same conditions that alkaline hydrolysis reaction.

The furfural was quantified spectrophotometrically using a wavelength of 305 nm, the maximum absorption wavelength determined by spectral sweeping in the UV–Vis regions, using a spectrophotometer Jenway model 6405.

## Results and discussion

### Moisture, morphology and elemental analysis of biomass

Moisture content percentage (%) is an important variable in the pectin extraction process. In the study samples, the moisture content percentage in wet base were 67%, 62%, 60% and 9% for mango, orange and tangerine peels and tamarind seeds, respectively. The SEM photomicrographs of the four study biomasses show significant differences in size and morphology (see Figure S1, support information). In the case of the mango peels (*Manguifera Indica L*.) (Figure S1a), it can be seen a mixture of fibrous agglomerations and spherical structures. The spheres range in diameter from 0.68 micron to 2 micron. In the orange peel (C*itrus sinensis*) (Figure S1b), thick agglomerated fibers could be seen (18.7 micron to 50 micron long with variable thicknesses).. Finally, On the other hand, tamarind seeds (*Tamarindus indica L.*) (Figure S1c), which had to be ground due to their original size, present a heterogeneous morphology in shape and size, rather use the term ranged between 3 to 30 micron structurestangerine peels (*Citrus reticulada L.*) (Figure S1d), showed a homogeneous matrix of tangled roots like fibers (structures with lengths above 50 micron).

Also, it was possible to identify the presence of salts in the biomasses by means of an elemental analysis (see Table S3, Support information). Salt content was analyzed since it could have influenced the solutions pH variations during pectin extraction with HCl and H_2_SO_4_. For the four biomasses, K was present, the largest amount contained in the tamarind seeds (1.74%w). The lowest amount was found in the orange peels with a 0.96%w (being almost twice smaller than the one in the tamarind seeds). Another element detected was Ca, which appeared only in the citruses (orange and tangerine) at 0.49%w for both enough. Finally, Mg was identified only in the tamarind peels in 0.66% in weight. The presence or absence of some salts in biomasses is attributed to biomass type, variety and cultivation region ^19^. The presence of salts in the solution may change the pH over the process duration and this may be detrimental to the quality of the extracted pectin.

All biomasses showed variations in pH values at the end of the process. However, no trend was observed with the pectin yields obtained in this study. Therefore, the effect of salts on the process cannot be identified. However, citrus biomasses were the samples with the greatest change in the pH value (2.2–2.9) and presented lower pectin yields than reported in other studies^4,38,40^. The tamarind seed was the biomass with the least effect on the pH values (2–2.2).

### Pectin yield determination

Pectin yields rather used ranged from 4 to 32.9%, the highest one was obtained from tamarind seeds using factorial design by evaluating the influence of temperature, time and catalyst in hydrolysis. As can be seen in Table 2, the yields were affected by the three study variables and by the type of biomass. For some factorial design conditions, mango and orange peels showed unacceptable yields to as biomasses that are rich in pectin (under 12%w in a dry base), as mentioned by Edwards et al.^12^ (2012).

**Table 2:** % Yield and DE pectin extraction from mango peels, orange peels, tangerine peels and tamarind seeds.

Exp	Mango peels		Orange peels		Tangerine peels		Tamarind seeds	
	Yield (%)	DE (%)	Yield (%)	DE (%)	Yield (%)	DE (%)	Yield (%)	DE (%)
1	8.5	42.1	8.55	54.45	12.45	57.48	12.6 ± 0	1.22
	± 0.5	± 0.2	± 0.35	± 0.35	± 0.45	± 0.48		± 0.03
2	4.5	47.45	8.3 ± 0.3	46.45	11.75	53.05	13.4 ± 0	32.43
	± 0.5	± 0.45		± 0.15	± 0.15	± 0.055		± 0.43
3	10.25	46.65	9.15	18.9	13.55	62.04	17.35	8.24
	± 0.25	± 0.035	± 0.15	± 0.1	± 0.25	± 2.9	± 0.15	± 0.14
4	6.55	40.37	9.7 + 0.2	57.5	13.65	66.07	18.85	30.41
	± 0.55	± 0.23		± 0.3	± 0.35	± 0.125	± 0.05	± 0.41
5	15.5	46.6	13.5 ± 0.3	54.2	14.15	55.25	21.5	52.1
	± 0.5	** ± 0**.1		± 0.2	± 0.15	± 0.245	** + 0**.4	± 0.1
6	14 ± 0	61.1	14.25	53.2	16.35	66.9	32.45	28.04
		± 0.1	± 0.25	± 0.8	± 0.35	± 0.1	± 0.45	± 0.06
7	17.1	35.6	16.9 ± 0.6	30.3	19.65	62.26	29.1	7.77
	± 0.1	± **0**.1		± 0.9	± 0.35	± 0.255	** + 0**.1	± 0.17
8	15.1	58.5	14.95	51.04	16.05	62 ± 0.1	31.45	49.05
	± 0.1	± 0.2	± 0.05	± 0.04	± 0.55		± 0.05	± 0.05

The maximum yields for the four biomasses were: mango peels 17.1% (Exp 7), orange peels 16.9% (Exp 7), tangerine peels 19.65% (Exp 7) and tamarind seeds 32.45% (Exp 6). For the first three biomasses the yields were enhanced with Exp 7 conditions, 80 °C for 60 min and HCl. Whereas for tamarind seeds the yield increased at 80 °C for 30 min and H_2_SO_4_ (Exp 6), a relative long period (Exp 8) of extraction would cause pectin thermal degradation^48^.

As can be seen the pectin yield results (Table 2) of citrus peels, different trends in values. The orange peel requires more time for protopectin (insoluble) to solubilize and increase the production of soluble pectin^48,49^. Pectin yields have been reported to be greater than those obtained in this study, using longer times. Reported pectin yields were: 25.8% (HNO_3_ and 100 min)^38^ and 29.3% (H_2_PO_4_, 120 min)^19^. The maximum yield for the extraction of pectin from tangerine peel was the same as that reported by Chen et al. (19.9%)^4^. The results of ANOVA are presented in Table S4 (Support information). The determination coefficient of yield of mango peels pectin, orange peels pectin, tangerine peels pectin and tamarind seeds pectin was 99.25%, 99.01%, 97.89% and 99.91%, respectively. The high value of R^2^, adj-R^2^ and pre-R^2^ (all biomasses) clearly stated that the relationship between the response and independent variables is well correlated (see Table S4, Support information)^8^. The higher model F-value and the associate lower *p*-values demonstrated that the developed model was significant.

**Figure 1:**
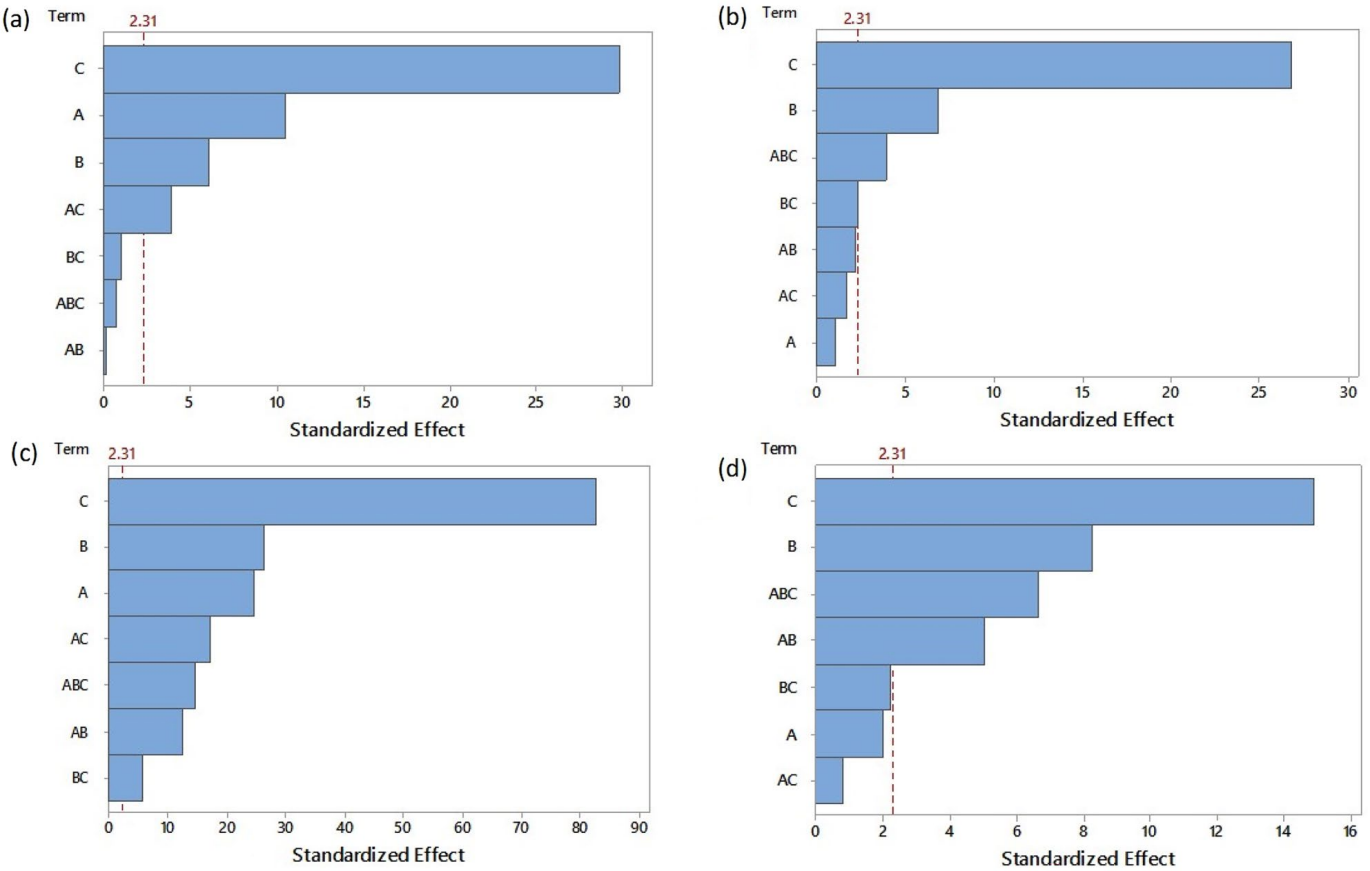
Pareto diagram for pectin extraction yield, (**a**) mango, (**b**) orange, (**c**) tamarind, and (**d**) tangerine (A = catalyst type; B = time and C = temperature).

Figure 1 shows the variance Pareto diagrams (ANOVA) of the statistical analysis of a factorial design 2^3^ in the pectin extraction process (yield, %), the sources: (a) mango peels, (b) orange peels, (c) tamarind seeds and (d) tangerine peels. The magnitude and order of the effects used, catalyst type, time and temperature (with a level of confidence of 0.05).

For all the cases, independently of the biomass used for the pectin extraction, the most important factor to increase the yield in acid hydrolysis was the temperature. For tamarind seed pectin, all the individual factors and interactions showed a statistic significance above 95%.

To evaluate the direction of the effects, standardized effect graphics were used; in Figure S2 (a, b, c, d) significant values (red squares) and not significant values (blue circles) were observed. Considering that the factors are: A (HCl and H_2_SO_4_), B (30 min and 60 min), and C (60 °C and 80 °C).

For mango pectin (Figure S2a), it could be seen that the catalyst (A), presented a negative standardized effect, whereas time (B) and temperature (C) had a positive standardized effect, meaning that the yield decreased when H_2_SO_4_ was used. Tamarind biomass presented the opposite behavior (Figure S2c), in which using H_2_SO_4_ increased the yield. The type of catalyst did not statistically influence the yield in the citrus orange peels (Figure S2b) and tangerine peels (Figure S2d) extractions. On the other hand, the yield was increased across the four biomasses when using high levels of time and temperature (60 min and 80 °C).

It has been found in previous studies that temperature was an important variable in the process of pectin extraction ^25^, as can be seen in the results. The yield increase due to temperature can be attributed to the enhancement of solubility and diffusion of the acid in the solid matrix at higher temperatures^39^. Also, higher temperature was shown to enhance solubility of protopectin.

When analyzing the time effects during the extraction the yield was increased when the higher level (60 min) was used. The longer period of contact between the solvent and the biomass’ solid matrix favored the diffusion and mass transfer^25,38^, which aids in the disruption of cell wall structure and the protopectin separation.

Finally, the type of catalyst was influenced by the type of biomass that was used. In the case of the orange, tangerine and mango peels the yield increased in relation to HCl, and in the case of the tamarind seeds in relation to H_2_SO_4_.

The analysis of the effect of the salt mineral presence, type and morphology on the process was studied using only the tamarind seed pectin (eight experiments). The selection was based on the fact, that this biomass showed the best yield at the lowest moisture content. Table 3 shows the absence or presence of potassium or calcium for each pectin. The presence of Ca and Al in the pectin is because the proportion of these elements increased with respect to total sample^19^. Consequently the Ca and Al could be detected by EDS. In the systems where there was a longer time and sulfuric acid was used in the extraction process, the salts were released from the pectin surface. For short times, they appear in the samples. This could have been because they were leached during the long time of exposure to the solvent during hydrolysis.

**Table 3:** Mineral content in tamarind seed pectin (weight %).

Experiments	S	Ca	CI	K	Al
1		0.2	46	0.54	0.1
^2^	1.84	2.10		0.35	
3			1.42	0.50	0.32
4	1.24				
5		0.22	1.11	0.44	
6	0.40	0.35		0.26	
7			0.97	0.43	
8	1 24				

**Figure 2:**
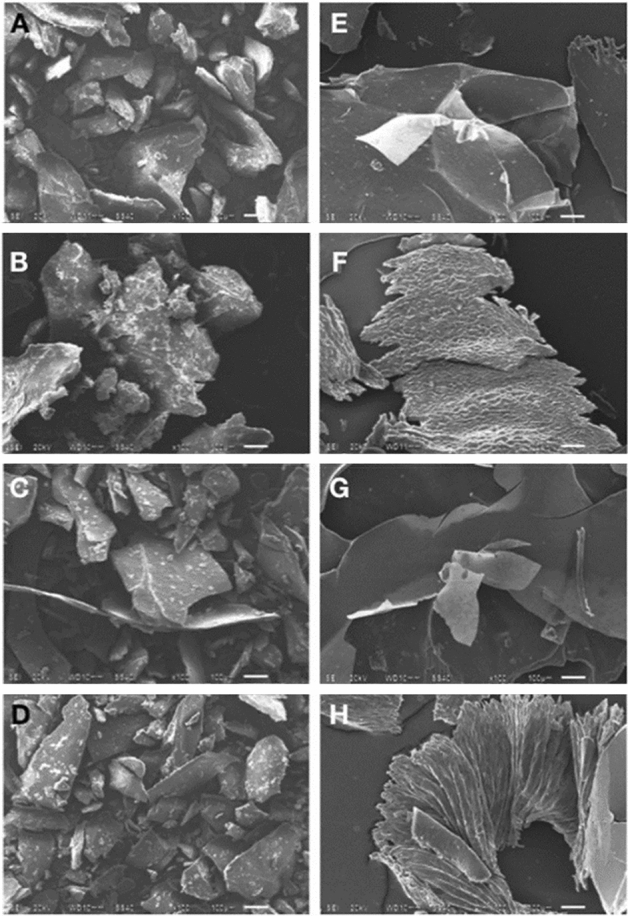
SEM images of tamarind seed pectin extracted, (**A**): Exp 1, (**B**): Exp 3, (**C**): Exp 5, (**D**): Exp 7, (**E**): Exp 2, (**F**): Exp 4, (**G**): Exp 6, (**H**): Exp 8.

In the case of the use of HCl, the experiments showed only the presence of potassium. Based on the morphological analysis (Fig. 2), several changes were observed based on the catalyst type. When using HCl, micrographies of A–D, showed heterogeneous structures and agglomerated lumps of different sizes. It is worth mentioning that in pectin C, a thinner morphology than in the other samples was observed. The SEM images using H_2_SO_4_ are showed in the micrographs E–H. Different forms are shown in these pectins; systems E and G presented structures shaped like fine, thin, and long sheets, which could be the most easily broken. It is worthwhile to mention that these sizes are larger than those of pectins with HCl. In system F, a pectin with a robust surface and variable sized porosity was obtained, also, it showed to be thicker than pectins E and G. Finally, pectin H was seen as fibrous tubular shaped agglomerated pectin fluctuating about 900 µm approximately.

Finally, the image and element analysis by SEM–EDS, lets points out that using HCl minimized the pectin yield, and that the time and temperature factors, did not influence the presence of calcium and the morphology in a significant way, opposite to the case when H_2_SO_4_ is used.

### Determination of Pectin DE

The DE determination was done by means of ATR-FTIR characterization; it was compared to commercial pectin (apple, Aldrich Sigma) and used as a reference. Figures S3, S4, S5 and S6 showed the peaks that are characteristic of the absorbance for each pectin in the study. In each case, the pectin was analyzed according to the experiment on Table 2. According to Gananasambandam and Proctor^50^ (2000), the peaks can undergo some shifting or changes in the samples due to a small difference in the structure and the composition of the molecules causing the spectral variations across the biomasses with different origin (Table S5). The main characteristic peaks are seen in Table S5, where the absorbances assignments corresponding to the wave length for pectins were shown, based on study references ^3,25,50^. The samples are: PM (mango pectin), PO (orange pectin), PTn (tangerine pectin) and PT (tamarind pectin).

Some changes were observed in the characteristic peak for stretching O–H, being samples E2, E5, and E7, the ones that showed lower intensities (Figure S3). Based on the vibrations at 1738 cm^−1^ and 1637 cm^−1^, there were some significant changes too. Nevertheless, the vibration at 1738 cm^−1^ had the most variations (esterified structure).

It was considered that the PO samples (Figure S4) were the ones that underwent fewer changes in the substances, that is, they presented better stability to the study variables in the extraction process.

For the PTn samples study, significant changes were observed in Figure S5. The intensity of the stretching O–H was affected in the vibration corresponding to the 3350 cm^−1^ wavelength. A larger magnitude prevailed for experiments E1 to E4 and for sample E5 a decrease compared the other samples was seen. In the same way, the peak corresponding to the vibration C–H (2929 cm^−1^), attributed to methyl ester^40^ underwent changes in intensity, mostly significant for E8. Also, it could be that for pectins (PTn), the vibrations at 1736 cm^−1^ and 1636 cm^−1^ were more stable to the other.

In the PT samples (Figure S6), the stretching corresponding to O–H (3329 cm^−1^) could be seen, they did not present any significant alterations. The same happened for the 2925 cm^−1^ peak_._ The most relevant changes were seen in the 1737 cm^−1^ and 1630 cm^−1^wavelenghts.

Generally, the comparison of the shiftings shown in PM, PO, PTn and PT, were more evident in the peak characteristic to the stretching O–H, representing a structural difference in the samples in the inter and intra molecular bond of the galacturonic acid spine. On the other hand, the range about 1500–800 cm^−1^ represented by each pectin’s ¨finger print¨, where different peptic substances can be distinguished by this region, showed a significant difference for each pectin extracted from the biomasses. These bonds are unique to a component and their interpretation can be difficult^21,51^.

To determine the % DE we took into account the absorbances of the characteristic peaks around the ranges of 1733–1738 cm^−1^ and 1630–1637 cm^−1^, which are associated respectively to esterified and non-esterified pectin^3,42,45^.

As it could be seen in Table 2, in the % DE value of the pectins from the study biomasses, high and low DE results were obtained. It was observed that the tamarind seeds, independently from the conditions of the factorial design, they all presented a low % DE excepting Exp 5. All tangerine peel pectins presented high % DE, whereas pectins extracted from mango and orange peels presented both low and high % DE. Pectin from tangerine peels had the highest % DE (Exp 4) with 67% compared all biomasses. Variations of high and low % DE in biomass can be attributed to controllable factors (conditions in the extraction process) and uncontrollable factors. An uncontrollable factor in this study was the degree of fruit ripening^48,52^. The ripening degree of the waste was not controlled. Samples were obtained in large quantities from the food industry and commerce.

Many studies have been carried out to measure the DE of pectin extracted from mango, orange, and tangerine peels. Nguyen et al.^49^ (2019), Girma and Worku^48^ (2016) and Banerjee et al. ^18^ (2016) found 52.6% DE, 72.17% DE and 69.1% DE of pectin from mango peel, respectively. The values reported were approximate to those obtained in this study (61.1% DE). However, the value of %DE may increase by using the sonification method as reported by Banerjee^18^ or increasing the process time^48^. For citrus pectin the % DE was reduced, which can be attributed to the type of acid used (organic or mineral) or/and process. DE values were 1.7–37.5% using the microwave process^21^.

The % DE showed an effect to the factorial design for the four types of biomass. Two of the biomasses presented a single pectin with high %DE (mango peels and tamarind seeds). The commercial grades of pectin generally suggested for food grade, have a DE value > 50% for the gelation in the presence of sugars. Some applications include fat replacers in spreads, salad dressings, ice cream, jams, jellies and emulsified meat products^8,21^. Typically, low DE pectin has higher value than the higher DE products. In many recent studies low DE pectin was found to be useful in low calorie and low fat products, since it requires calcium for gelling and little or no sugar. Low DE pectin was also found to be beneficial in lowering serum cholesterol level in humans ^18^.

In Figure S7, we can see the Pareto graphics for the analysis of the effect each %DE study variable had. Mango peel pectin, Figure S7a, showed the most significant effect with the interaction catalyst type- temperature; whereas orange peel pectin showed (Figure S7b), the %DE value showed a bigger effect regarding the interaction catalyst-time. Pectin extracted from tamarind seeds (Figure S7c), showed sensitivity in the %DE based on the interaction of the three factors (catalyst type-time–temperature). Finally, tangerine peel pectin (Figure S7d) showed that the %DE turned out to be more sensitive regarding time in the hydrolysis process.

Figure S8 shows standardized effects graphics, showing the following results. Figure S8a, showed that %DE for mango pectin, the individual effects such as catalyst type and temperature (H_2_SO_4_ y 80 °C), increased the % DE value. In the case of time, it is established that the longer the time (60 min) galacturonic acid undergoes de-esterification. Orange pectin (Figure S8b) and tamarind pectin (Figure S8c), showed the same tendency as in the previous case. Nevertheless, tangerine pectin (Figure S8d), was the biomass that reached the highest % DE values using the catalyst H_2_SO_4_ and longer time, favoring % DE. The temperature did not show a meaningful statistical tendency in the % DE values in this last case.

### Thermogravimetric properties of pectin

The weight loss in pectin thermograms reflects the variation on sample degradation in accordance with the % DE difference (Figure S9). The thermal degradation of pectin (tamarind, tangerine and mango) displays a characteristic three-step thermal degradation, in contrast with orange pectin that shows a one-step degradation. The first gradient in weight (%) is attributed to the moisture content present in the pectin^52^. For citrus pectin the have 3.3%, mango pectin 4% and tamarind pectin 5.3%.

Pectin presented a good control of the moisture content during the storage inhibits the growth of microorganisms, which directly influences the quality of pectin. The established maximum limit for moisture in quality pectin is 10%^19^. In pectin samples with high DE, a higher thermal stability up to 338 °C is observed. The higher thermal stability can be attributed to galacturonic acid de-esterification, a reaction that precedes to occur before the glyosidic bond can be degraded.

### Furfural production

The furfural production was evaluated using the pectin extracted from experiment 8 conditions (H_2_SO_4_, 60 min at 80 °C). These conditions agree with other studies reported in the literature^45,53^. The following sections discuss the two furfural production pathways and the impact of catalyst and the reaction time over the furfural concentrations.

#### Alkaline Hydrolysis

**Figure 3:**
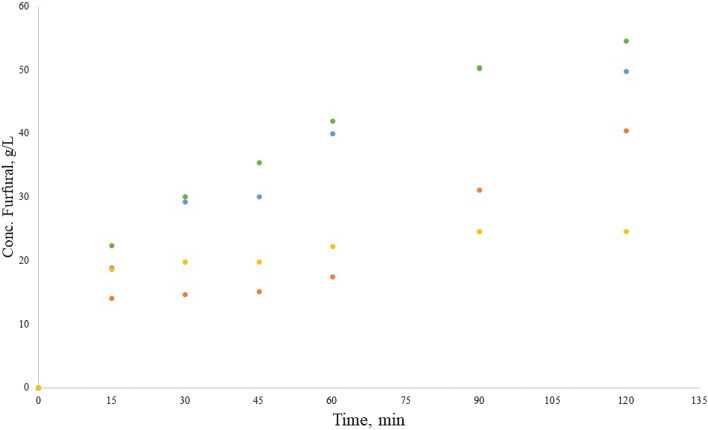
Furfural production in the hydrolysis reaction with Ca(OH)_2_, from orange pectin (blue circle), tangerine pectin (green circle), mango pectin (orange circle) and tamarind pectin (light orange circle).

The two types of alkaline catalysts here studied, Ca(OH)_2_ and CaCl_2_ were selected due to low cost, abundance and low toxicity^54,55^. In Fig. 3, the furfural concentrations obtained from Ca(OH)_2_ as catalyst are displayed, it can be observed that the tangerine pectin has the highest DE among all the samples. The tangerine pectin displays the highest furfural formation at all times when compared to the rest of the samples, the maximum concentration of furfural (54.7 g/L) was achieved at the end of the reaction time 120 min. Orange and tangerine pectin had a similar behavior during the initial stages, they reached a maximum concentration at 90 min. Mango and tamarind pectins showed lower furfural conversions than the citrus pectins, this could be due to the cleavage of the bond between galacturonic acid and the ester instead of the glycosidic bond among the galacturonic chain; by not breaking the glycosidic bond the available monomers for furfural production is reduced, which is in agreement with the results published by López-Mercado et al.^3^ (2018).

**Figure 4:**
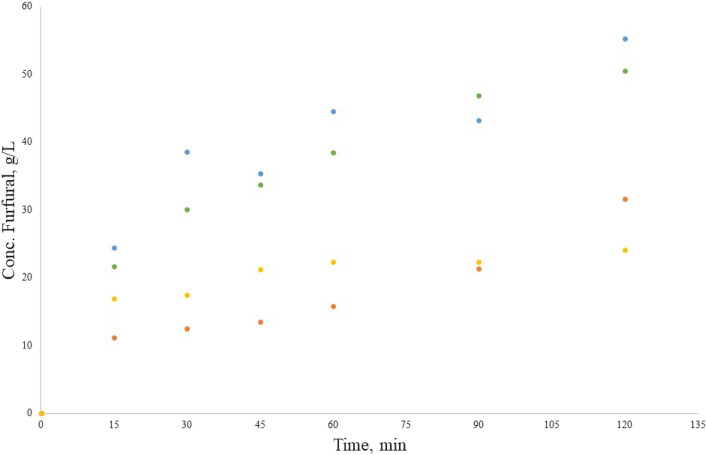
Furfural production in the hydrolysis reaction with CaCl_2_, from orange pectin (blue circle), tangerine pectin (green circle), mango pectin (orange circle) and tamarind pectin (light orange circle).

Figure 4 presents the furfural concentrations (g/L) obtained from the pectin alkaline hydrolysis with CaCl_2_ as catalyst. The orange pectin sample generated the highest furfural concentration of 55.3 g/L at 120 min, followed by tangerine pectin with 50.5 g/L at 120 min, then mango pectin obtained 31.6 g/L at 120 min, and lastly tamarind pectin with 24.1 g/L at 120 min.

#### Alkaline Maillard reaction

Maillard reaction can be divided in three stages: pectin de-polymerization, Amadory rearrangement and Schiff base formation. Every stage of the reaction is strongly dependent on temperature, pH and type of reactant (i.e. monosaccharide and amino acid employed)^56^. The Maillard reaction is an alternative way to produce furfural that has been reported in acidic conditions, in the present work the Maillard reaction is performed in an alkaline environment achieving higher concentrations than the acidic Maillard reaction previously reported^3^.

**Figure 5:**
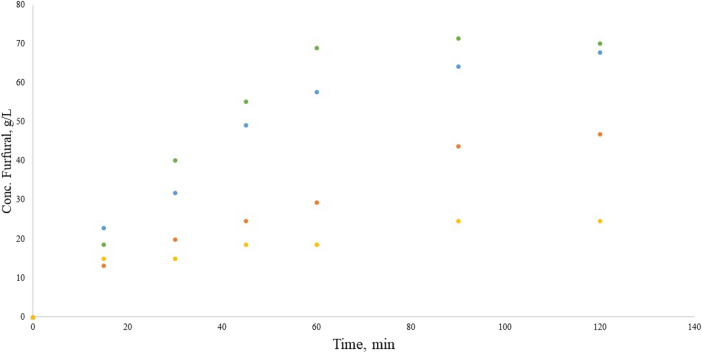
Furfural production in the Maillard reaction with Ca(OH)_2_, from orange pectin (blue circle), tangerine pectin (green circle), mango pectin (orange circle) and tamarind pectin (light orange circle).

Figure 5 displays the furfural production with Ca(OH)_2_ as catalyst in the Maillard reaction. The highest furfural concentration was achieved with tangerine pectin that reached a maximum (70.3 g/L) at 90 min, followed by the orange pectin with a maximum (67.9 g/L) at 120 min, mango pectin maximum (46.9 g/L) is found at 120 min and lastly the tamarind pectin had its maximum furfural concentration (24.7 g/L) at 90 min. The higher furfural concentrations are observed in the citrus pectins, such behavior could be due to the presence of organic acids in the solution formed as byproducts during the reaction from decomposition reactions of polysaccharide^57^. The acid solution improve the base Shiff formation hence the furfural production^56^.

Maillard reaction catalyzed with Ca(OH)_2_ seemed to reached a plateau behavior by the end of the reaction. The orange pectin was displaying the slowest transition to this behavior. The stagnant furfural concentration could be due to the presence of galacturonic acid in the reaction or an increased stability of furfural in the reaction mixture.

**Figure 6:**
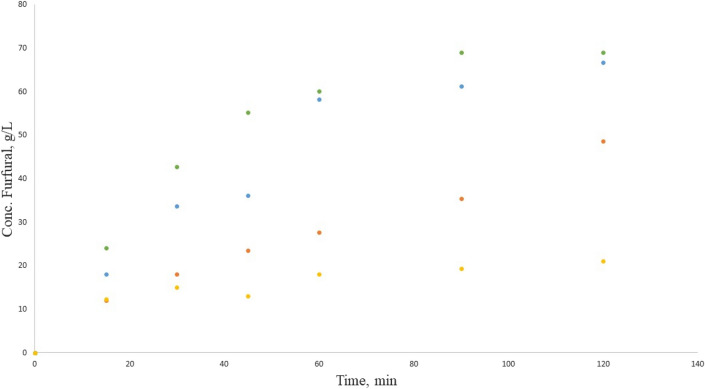
Furfural production in the Maillard reaction with CaCl_2_, from orange pectin (blue circle), tangerine pectin (green circle), mango pectin (orange circle) and tamarind pectin (light orange circle).

Figure 6 contains the furfural concentrations through time when CaCl_2_ is used as catalyst in the Maillard reaction. The trend observed is similar to that observed in Fig. 6, with similar values which suggested that both catalysts promote the glycosidic bond cleavage between the galacturonic acid monomers, that one released reacts with the amino group from lysine and develops the reaction cascade that leads to furfural formation.

## Conclusion

Pectin is a compound of high added value, and its high production in Mexico is very important for diverse uses. Based on a factorial design (temperature, time and catalyst) four second generation biomasses were analyzed, obtaining 32.45% on dry base or 31.3% on wet base of pectin for Exp 6 (H_2_SO_4_, 30 min and 80 °C) was generated for the tamarind seeds. The yield value obtained is higher than the reported in other studies.

Also, pectin extracted from tamarind seeds in Exp 6 showed a low DE (28.04%). It is worth mentioning that in system Exp 8 (H_2_SO_4_, 60 min and 80 °C) with 49.0 5% DE was produced. The differences in both systems (Exp 6 and Exp 8) are relatively significant in % DE and morphology evaluated by SEM. The differences were due to exposed time in hydrolysis, where the galacturonic acid underwent de-esterification the longer the time.

The tangerine peel biomass presented a higher resistance in the de-esterification of the galacturonic acid at prolonged times compared to other biomasses, resulting in a pectin with 66.9% DE at temperature of 60 °C, H_2_SO_4_ and 60 min.

Even when the factorial design exposed the independent variables effect, the temperature showed to be the one with the most statistical significance to increase the yield of the extracted pectin, no matter the extraction source. For the % DE, different conclusions are presented for each second generation biomass type.

This type of pectin could be used in intermediaries to biofuels production (furfural) or biogas. The biogas production benefits with a low % DE for pectin between the room temperature to 342 ºC range, resulting that for its thermal decomposition high % DE pectin require 1.2 times the temperature required for low % DE pectin, pointed out in the TGA.

We found no evidence of correlation between the furfural production and the % DE which opens a wide new field to the use of low % DE that have been neglected due to the lack of application in the food and cosmetic industries. The application of the Maillard reaction in the alkaline hydrolysis media improved the furfural production in all pectins except tamarind pectin, it reached a 54% increase in the mango pectin when CaCl_2_ was used as catalyst. The Highest furfural production through the alkaline Maillard reaction was favored using tangerine pectin and Ca(OH)_2_ reaching a maximum of 71.8 g/L at 90 min, which represents an increase of 42% when compared with the Ca(OH)_2_ hydrolysis at the same time. In general, the alkaline Maillard reaction achieved higher concentrations of furfural when compared to alkaline or acidic hydrolysis and even the acidic Maillard reaction. The alkaline Maillard reaction achieved higher concentrations in shorter times in the case of citrus pectins which could suggest an accelerating effect over pectin de-polymerization, Amadory rearrangement and Schiff base formation are stages of the Maillard reaction resulting in furanic compounds. These stages are greatly influenced by temperature, pH and the nature of the pectine and the protein involved. The present work demonstrated that the pH has a very strong effect over furfural production through the Maillard reaction. Citrus pectins developed higher furfural concentrations with lower pH (~ 2) while mangoes pectin and tamarind pectin got lower concentrations with higher pH (~ 3), we believe this pH difference has a positive effect during the base Shiff formation in the furfural formation process.

## Supplementary Information

Supplementary Information.

## Data Availability

The datasets generated and/or analyzed during the current study are not publicly available due copyright, we declare all data have being generated and gathered in agreement with our institutions authorization for research and publication purposes. Available from the corresponding author on reasonable request.
