# Supplementary material for: Evaluation of pectin extractions and their application in the alkaline Maillard reaction
Source: Sci Rep. 2022 Nov 18;12:19834. doi: 10.1038/s41598-022-22002-9 (PMC9674671; doi:10.1038/s41598-022-22002-9)
Supplement: Supplementary file 1 — Supplementary Information. [file 41598_2022_22002_MOESM1_ESM.docx]

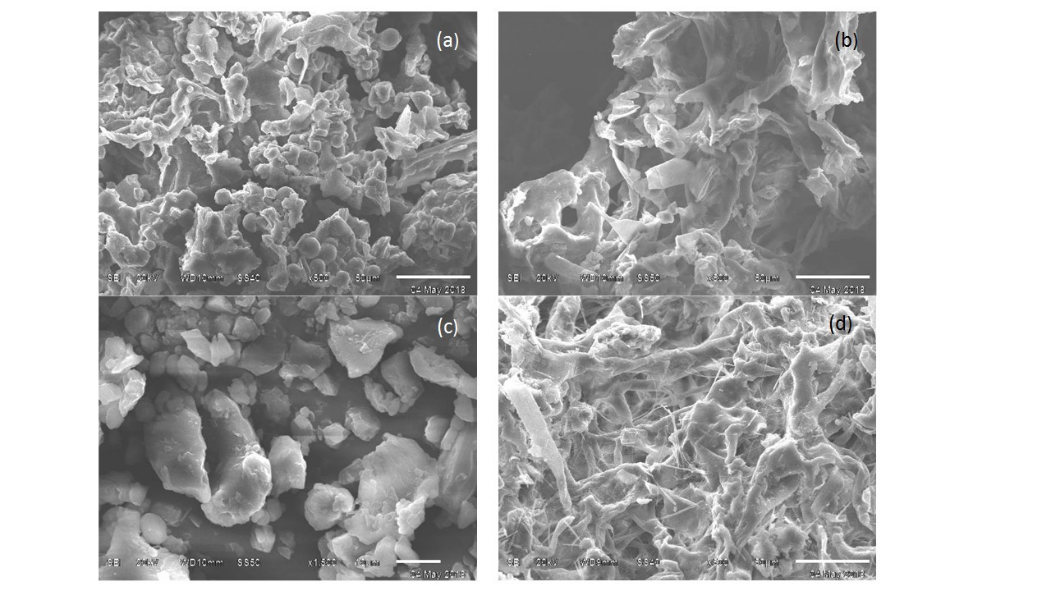


***Figure S1.***  *SEM images of biomass of (a) mango peel, (b) orange peel, (c) tamarind seed, and (d) tangerine peel.*


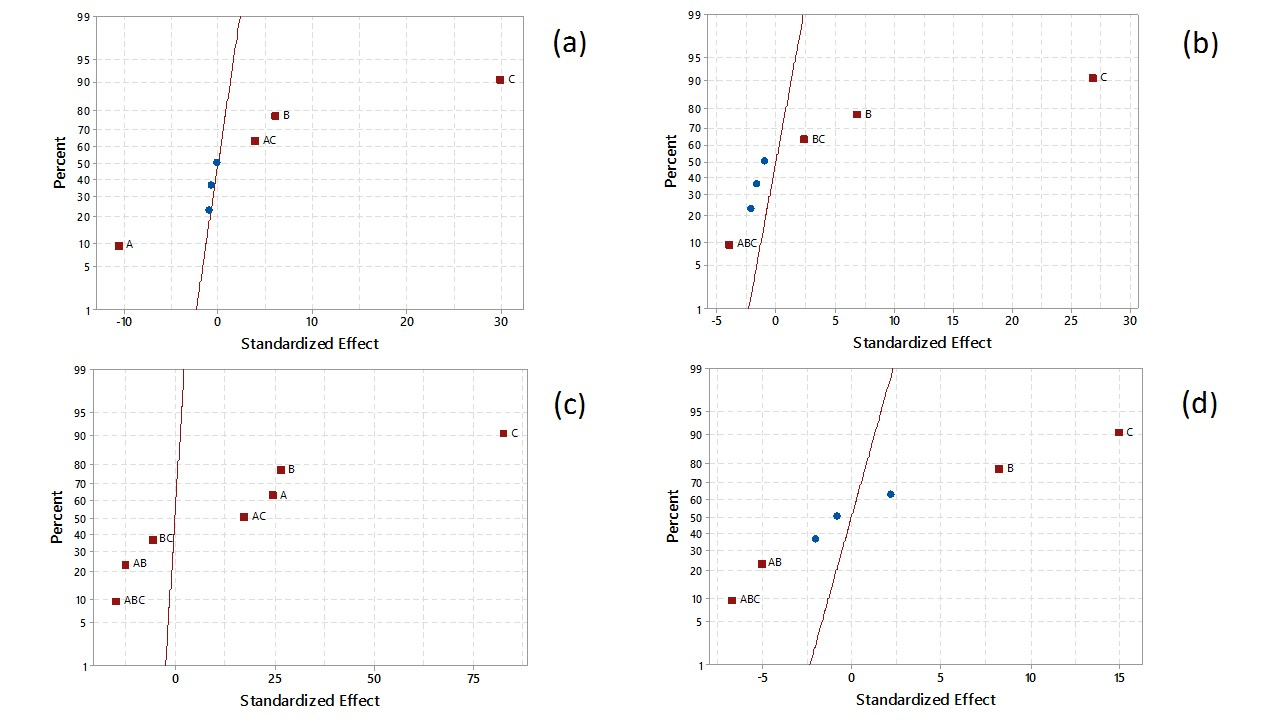


***Figure S2.*** *Standardized effect diagram for pectin extraction yield, (a) mango, (b) orange, (c) tamarind, and (d) tangerine (A= catalyst type; B= time and C=temperature).*

*
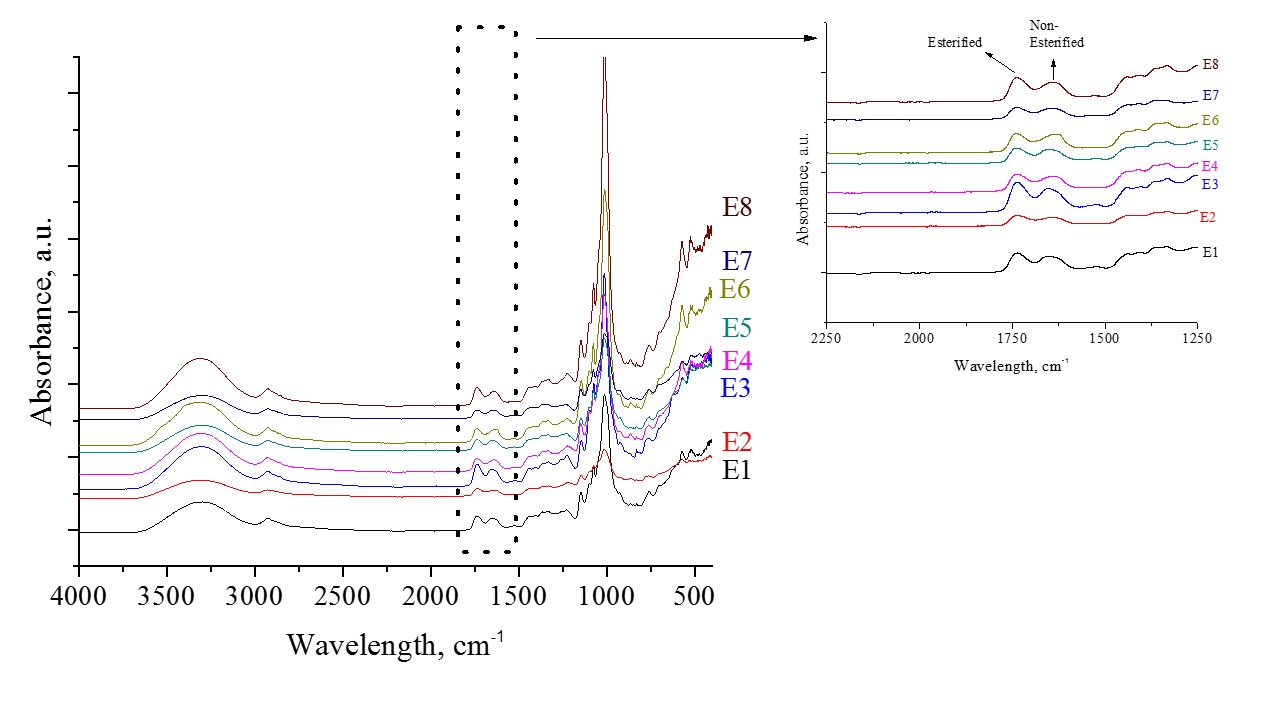
*

***Figure S3.*** *ATR-FTIR spectrum of mango peel pectin extracted under factorial design conditions.*


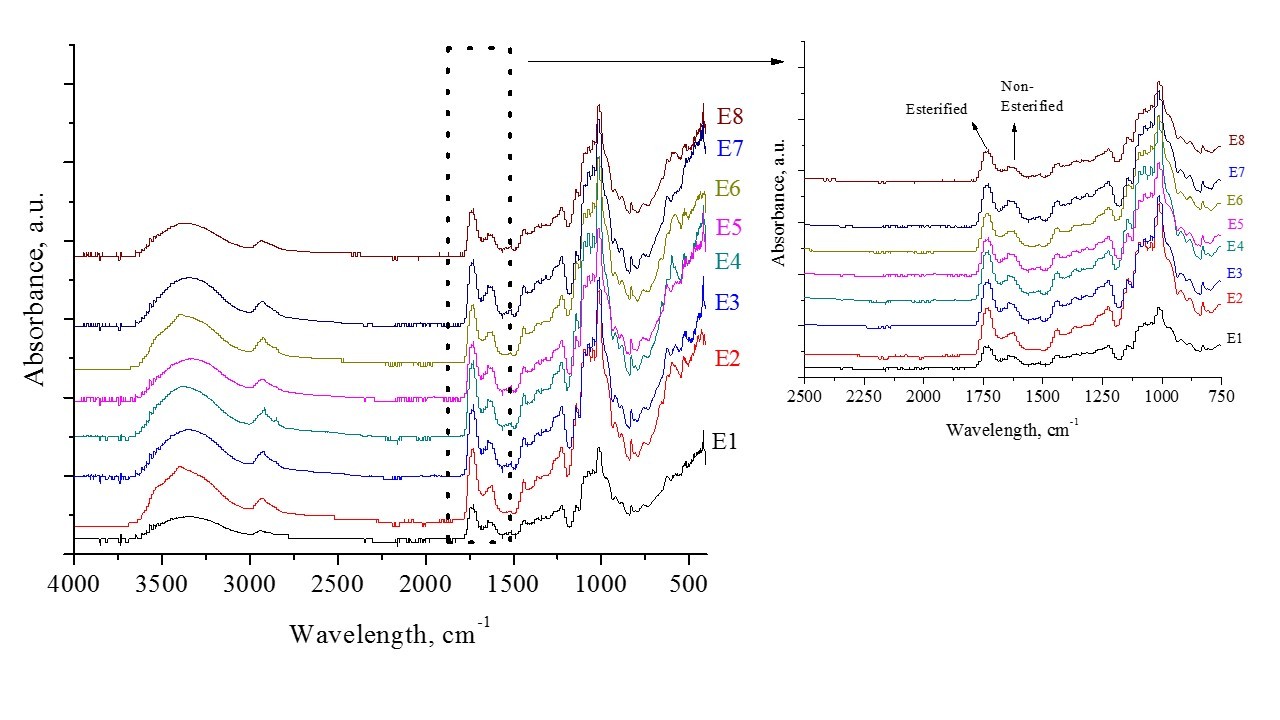


***Figure S4.*** *ATR-FTIR spectrum of orange peel pectin extracted under factorial design conditions.*


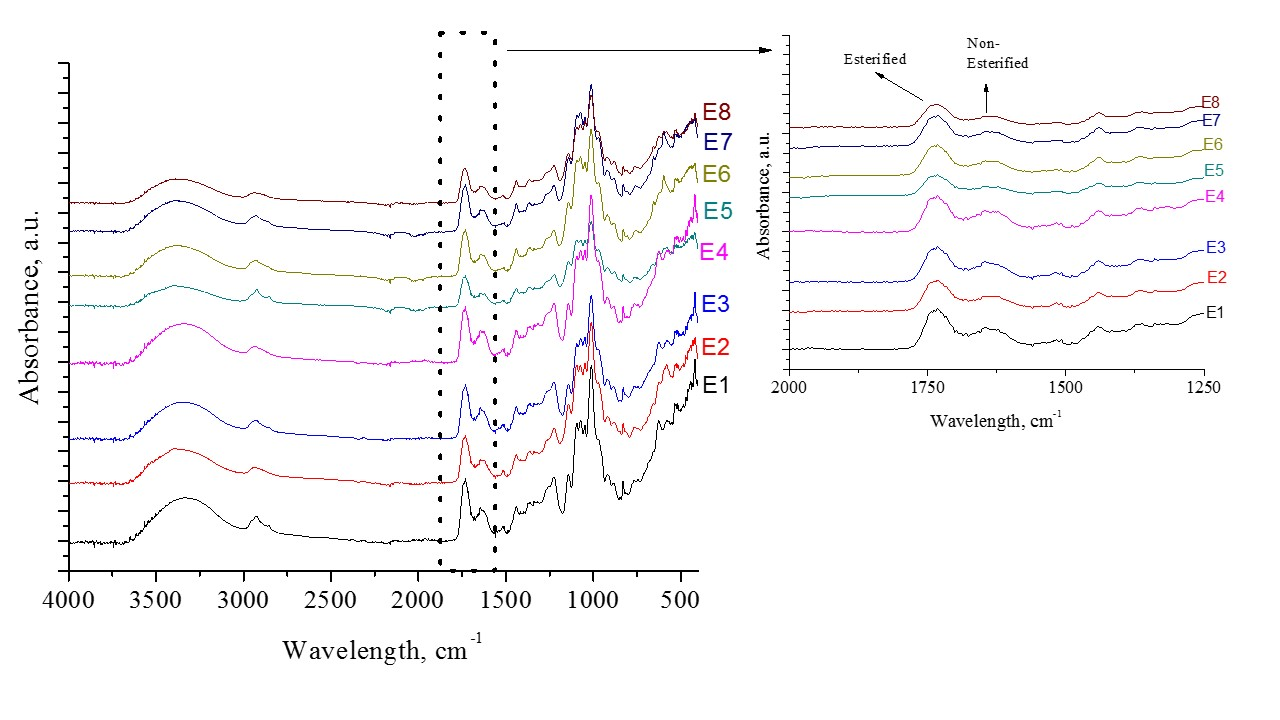


***Figure S5*** *ATR-FTIR spectrum of tangerine peel pectin extracted under factorial design conditions.*


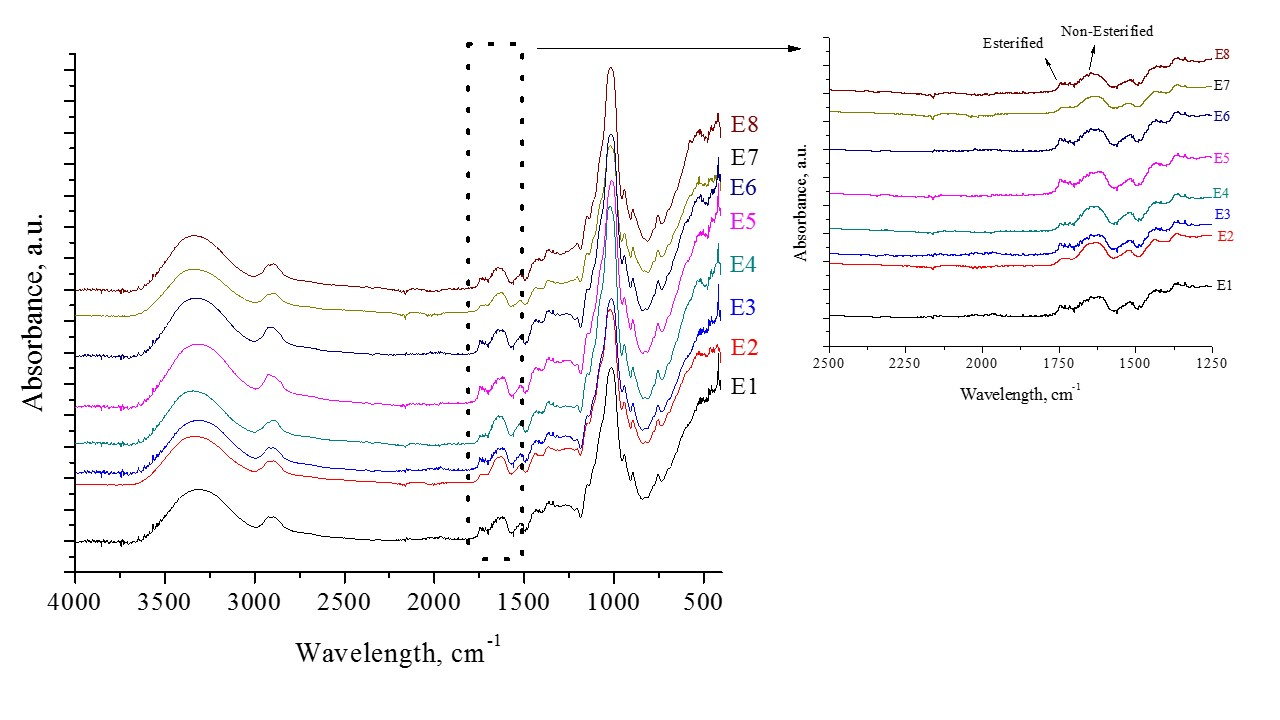


***Figure S6.*** *ATR-FTIR spectrum of tamarind seed pectin extracted under factorial design conditions.*


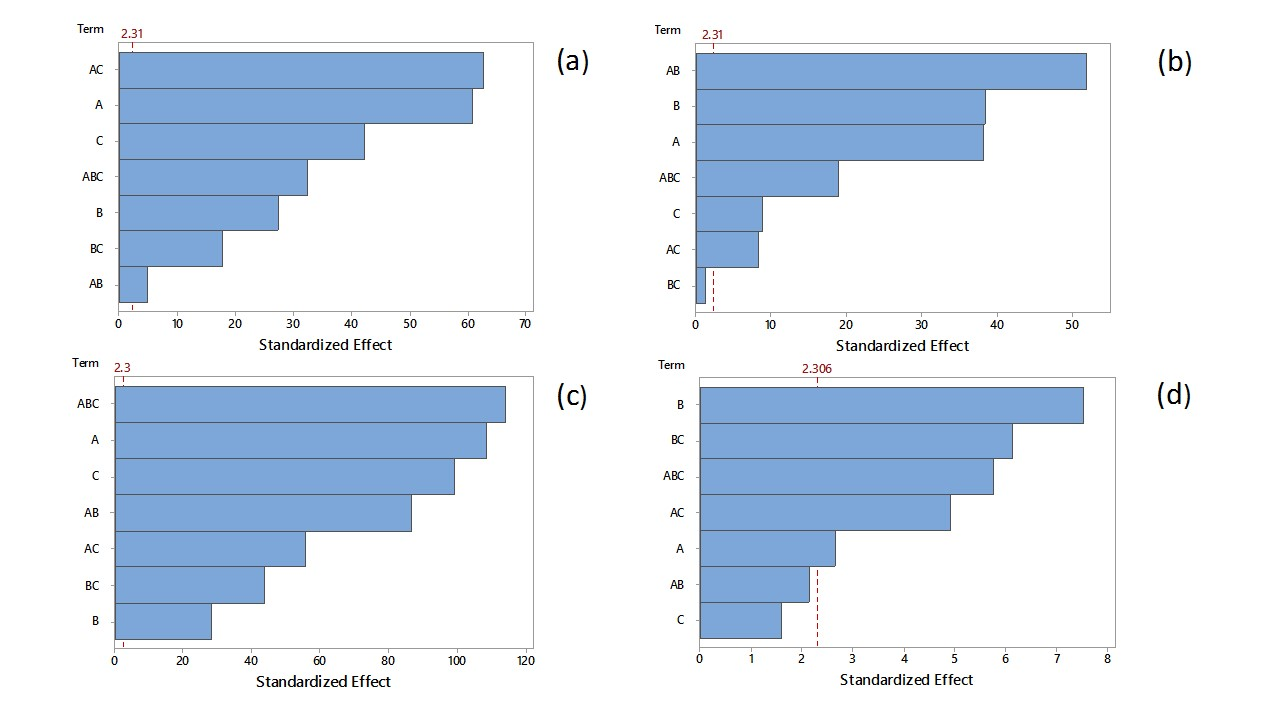


***Figure S7.*** *Pareto Diagram for pectin DE,* ***(****a) mango, (b) orange, (c) tamarind, and (d) tangerine (A= catalyst type; B= time and C=temperature).*


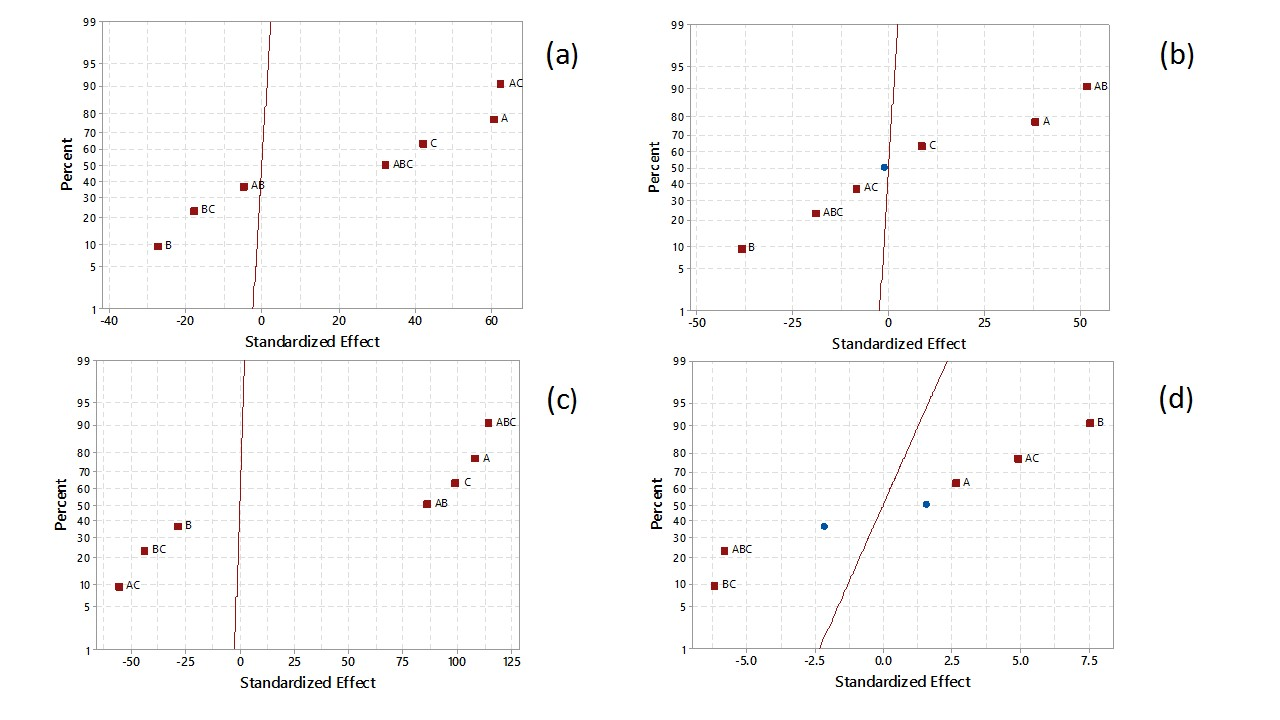


***Figure S8.*** *Standardized effect diagram for pectin DE, (a) mango, (b) orange, (c) tamarind, and (d) tangerine (A= catalyst type; B= time and C=temperature).*


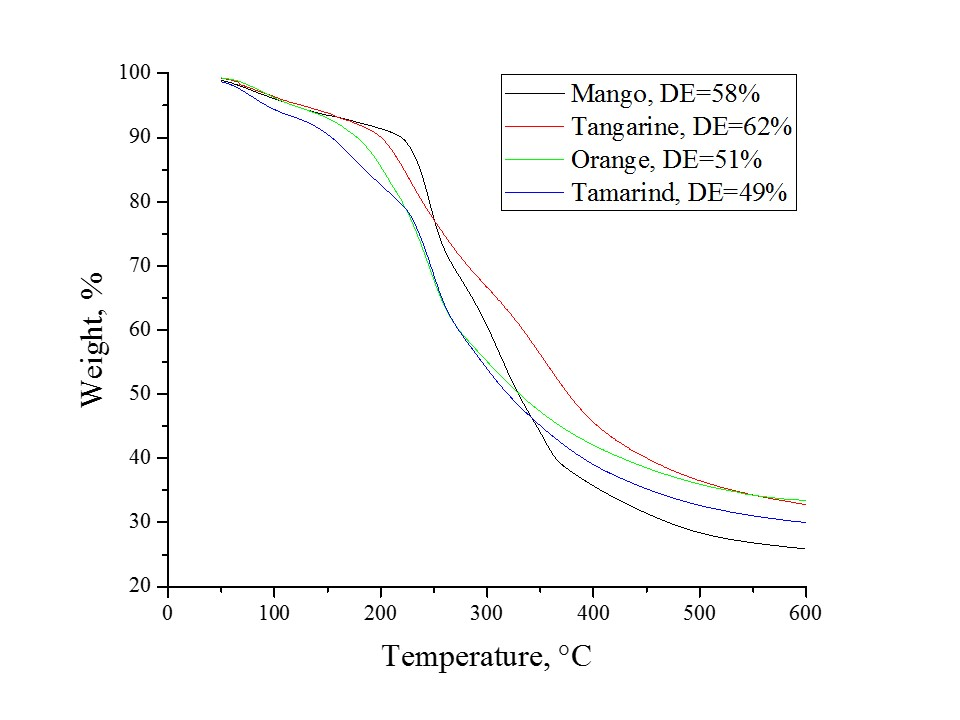


***Figure S9.*** *TG analysis of pectin extracted from biomasses under factorial design conditions of Exp 8.*

***Table S1.*** *Design and experimental results of 2^3^ full-factorial design.*

*
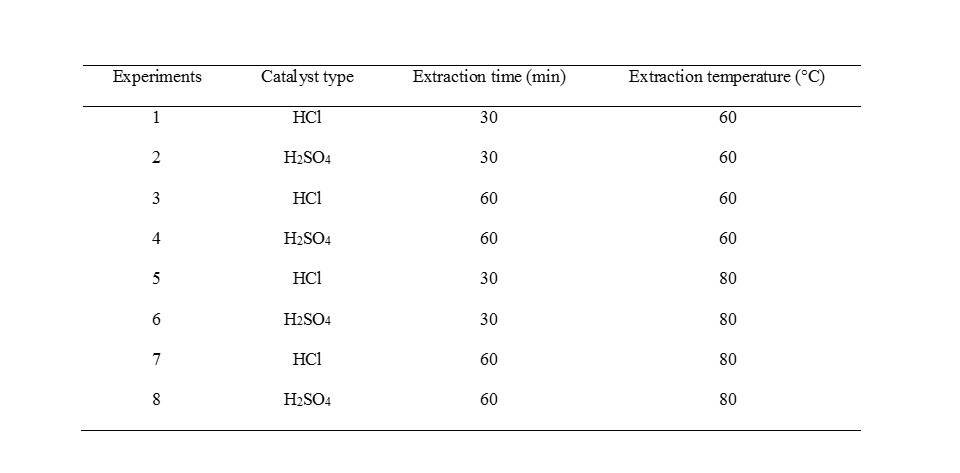
*

***Table S2.*** *Factorial Design variables and levels, where (-1) is the lowest level, and (+1) the upper one.*

*
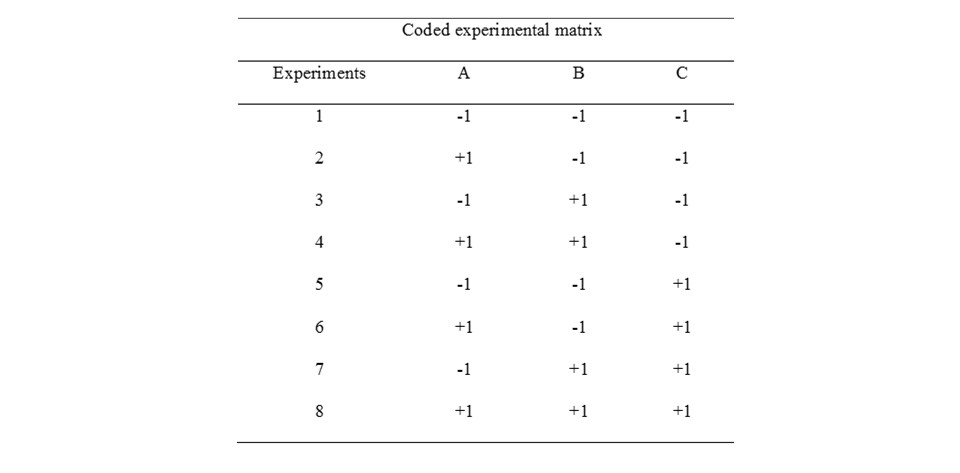
*

***Table S3.*** *Mineral content in biomass (weight %).*

*
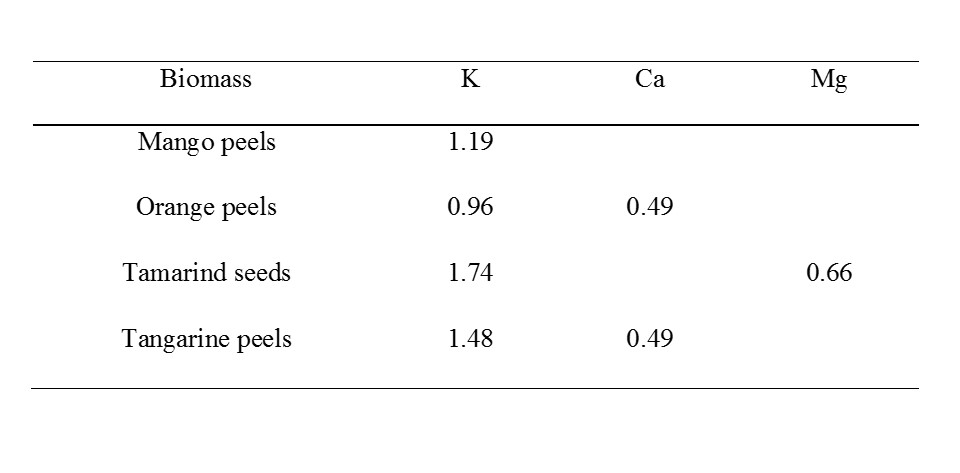
*

***Table S4.*** *The results of analysis of variance (ANOVA) of biomass pectin yield.*


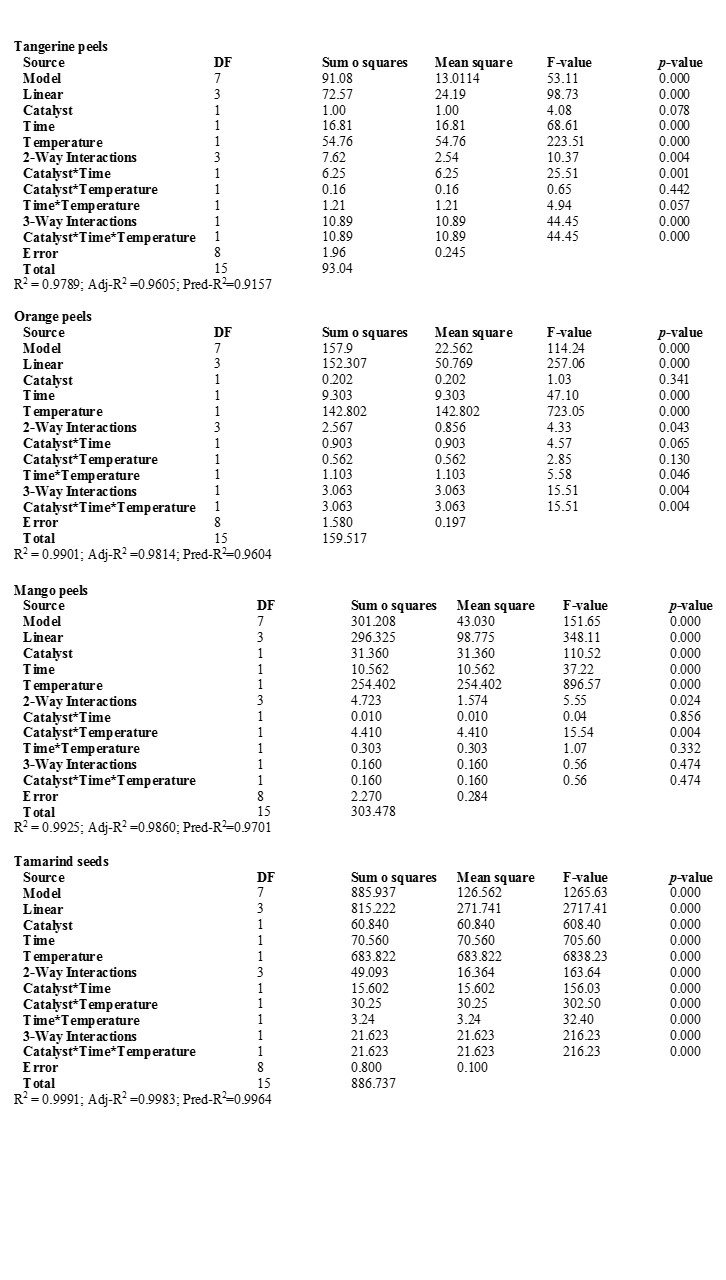


***Table S5.*** *Assignments of ATR-FTIR of pectins from differences second generation biomasses.*

*
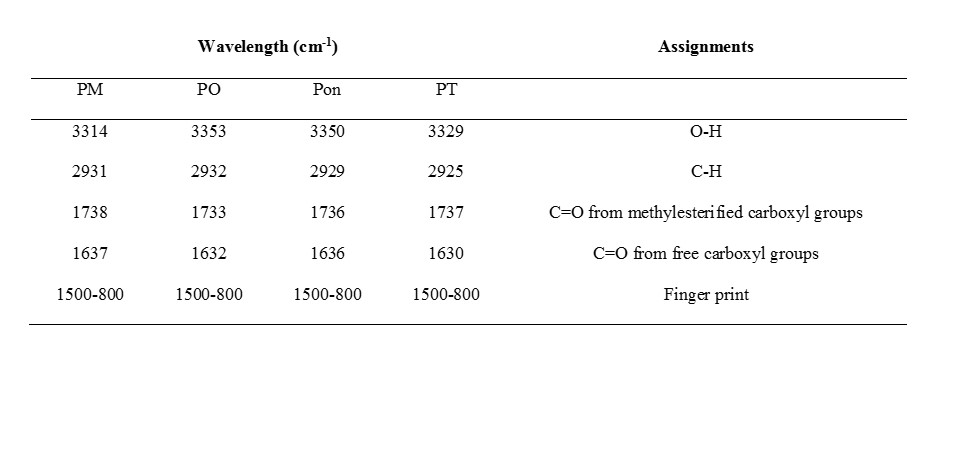
*
